# S1P/S1PR signaling pathway advancements in autoimmune diseases

**DOI:** 10.17305/bb.2023.9082

**Published:** 2023-12-01

**Authors:** Jianbin Li, Yiping Huang, Yueqin Zhang, Pengcheng Liu, Mengxia Liu, Min Zhang, Rui Wu

**Affiliations:** 1 Department of Rheumatology and Immunology, The First Affiliated Hospital of Nanchang University, Nanchang, China

**Keywords:** Sphingosine-1-phosphate (S1P), sphingosine-1-phosphate receptor (S1PR), S1P/S1PR signaling pathway, autoimmune diseases, research progress

## Abstract

Sphingosine-1-phosphate (S1P) is a versatile sphingolipid that is generated through the phosphorylation of sphingosine by sphingosine kinase (SPHK). S1P exerts its functional effects by binding to the G protein-coupled S1P receptor (S1PR). This lipid mediator plays a pivotal role in various cellular activities. The S1P/S1PR signaling pathway is implicated in the pathogenesis of immune-mediated diseases, significantly contributing to the functioning of the immune system. It plays a crucial role in diverse physiological and pathophysiological processes, including cell survival, proliferation, migration, immune cell recruitment, synthesis of inflammatory mediators, and the formation of lymphatic and blood vessels. However, the full extent of the involvement of this signaling pathway in the development of autoimmune diseases remains to be fully elucidated. Therefore, this study aims to comprehensively review recent research on the S1P/S1PR axis in diseases related to autoimmunity.

## Introduction

Autoimmune disorders result in damage to organs or the entire body system due to a dysregulation of the immune system and subsequent destruction of cells and tissues [1, 2]. Although autoimmune diseases were rare in the past, recent epidemiological studies indicate a prevalence of 3%–5% in the population, with increasing incidence rates posing significant risks to patients’ lives and overall well-being [3]. While the exact causes and mechanisms behind these diseases remain unclear, extensive research has shed light on the crucial role of the sphingosine-1-phosphate (S1P)/S1P receptor (S1PR) signaling pathway in the inflammatory response associated with autoimmune disorders [4]. Consequently, the therapeutic potential of targeting S1PRs for the treatment of autoimmune and inflammatory conditions has garnered considerable attention from the scientific community [5]. This article provides an overview of the current research advancements pertaining to the involvement of the S1P/S1PR axis in autoimmune diseases.

**Figure 1.**
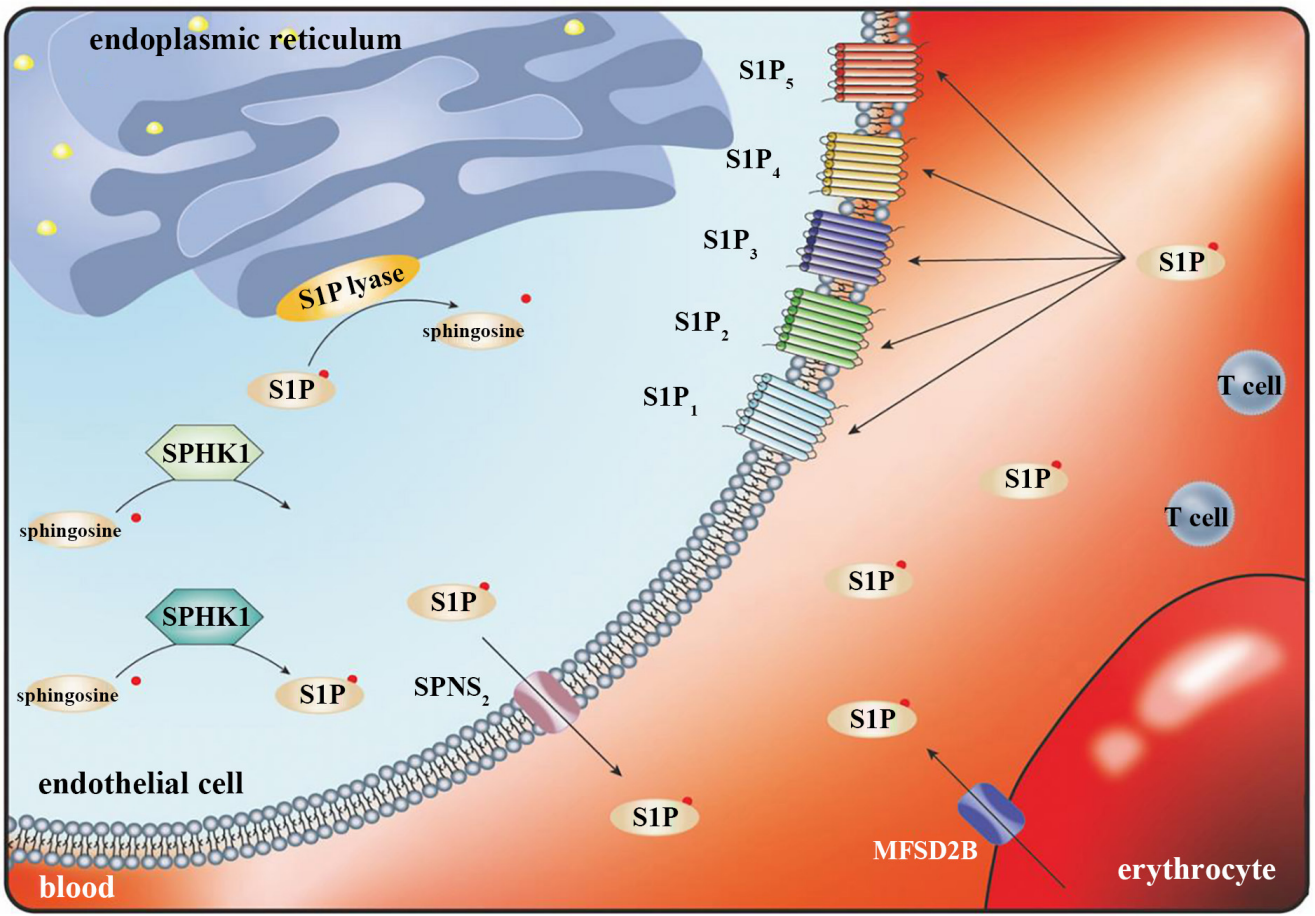
**Schematic summary of the current view of S1P signaling pathways.** S1P: Sphingosine-1-phosphate; SPHK1: Sphingosine kinase 1.

## Physiopathological role of S1P and S1PRs

S1P, a member of the sphingolipid family, is a biologically active lipid mediator that undergoes conversion from ceramide to sphingosine by ceramidase catalysis [6]. Subsequently, sphingosine is phosphorylated to S1P by sphingosine kinase 1 (SPHK1) and sphingosine kinase 2 (SPHK2) [7]. SPHKs primarily exist in two distinct isoforms, namely, SPHK1 and SPHK2, which are associated with the synthesis of S1P in extracellular and intracellular compartments, respectively [8]. S1P functions as a pleiotropic lipid mediator that regulates the activities of numerous cell types. However, in mammalian systems, S1P is found predominantly in blood and lymphatic fluid under normal physiological conditions. Erythrocytes, platelets, and endothelial cells are the primary sources of circulating S1P [9], although mast cells and platelets can also produce excessive amounts of S1P in an inflammatory or pre-thrombotic state [10]. Erythrocytes lack S1P-degrading enzymes but express the specific S1P transporter Mfsd2b [11]. Deficiency of Mfsd2b leads to a 50% reduction in plasma S1P concentration. Conversely, endothelial cells express another specific S1P transporter, Spns2, which contributes to the maintenance of circulating S1P concentrations. Deficiency of Spns2 results in a 40% decrease in S1P concentration in plasma and 80% decrease, indicating that lymphatic endothelial cells predominantly maintain S1P in lymph [12, 13]. Platelets also lack S1P-degrading enzymes but express the S1P transporter Mfsd2b, although their contribution to circulating S1P concentrations is minimal, as evidenced by the absence of changes in plasma S1P concentration in platelet-deficient mice [14]. In tissues, S1P is rapidly degraded by S1P lytic enzymes, resulting in a marked contrast between high intravascular S1P concentrations and low tissue S1P concentrations [15]. The S1P_1_ receptor plays a crucial role in regulating the trafficking of T and B cells from secondary lymphoid organs into the lymphatic system and bloodstream by facilitating receptor-mediated chemotaxis toward the highest S1P concentration gradient in blood/lymph. This S1P gradient is essential for maintaining physiological tissue homeostasis, by directing cells from the low S1P environment of tissues to the high S1P environment of circulating fluid [16]. Disruptions in the S1P gradient can alter the migratory behavior of immune cells and other cell types. Changes in the local S1P gradient, which can be triggered by local injury and subsequent inflammatory responses, modulate the activity and migratory behavior of immune cells and vascular wall cells. From a pathophysiological perspective, local alterations in S1P gradients support immune system responses and enhance local defense and healing processes. There is speculation that the S1P gradient might play an important role in lymphocyte egress [17]. Initially, S1P was defined as an intercellular second messenger [18, 19] that interacts with intracellular targets, such as inhibin 2 and tumor necrosis factor receptor-associated factor 2. The physiological significance of intracellular S1P signaling was unknown at the time. However, subsequent research has revealed the existence of five high-affinity receptors, known as S1PRs, which are a class of G protein-coupled receptors located on cell membranes. Five S1PR subtypes have been identified, namely, S1P_1_, S1P_2_, S1P_3_, S1P_4_, and S1P_5_ (Figure 1). S1PRs are characterized by tissue-specific expression and partially synergistic and antagonistic functions. Nearly all cell types in the body appear to express S1PRs. The typical receptor-mediated effects of S1P encompass cell proliferation, migration, and apoptosis [20]. Notably, S1PRs are highly expressed in immune cells, including neutrophils, dendritic cells, natural killer cells, and macrophages, and may regulate inflammatory diseases and wound healing processes, as well as various cellular trafficking events involving hematopoietic cells and lymphocytes. S1P serves a critical role in immune cell responses, such as B cell migration and their interaction with T cells. Furthermore, it influences the localization of immune cells, such as natural killer cells, within lymph nodes, and modulates their response to interferon-gamma [21]. Consequently, the S1P/S1PR axis is implicated in immune-mediated diseases and has been proposed as a potential target for the prevention or treatment of certain conditions.

S1P_1_ is expressed in various tissues and organs, including the brain, lungs, liver, heart, and spleen. It serves as a crucial receptor on the surface of lymphocytes, mediating their homing [22]. Under normal circumstances, S1P_1_ expression is upregulated in lymphoid organs due to the influence of lymphocytes, and it is also expressed in oligodendrocytes and neurons in the central nervous system (CNS) [23]. In mesenchymal stem cells, its expression regulates cell migration, proliferation, differentiation, and survival [24]. S1P_1_ is involved in vascular system development and is necessary for maintaining endothelial cell barrier integrity and vascular permeability [25]. Consequently, its expression is high in differentiated endothelial cells [26] and is further upregulated in inflammatory environments. S1P_1_ exerts anti-inflammatory effects by inhibiting the expression of pro-inflammatory adhesion molecules 2–4 on leukocytes and limiting cytokine production [4]. Additionally, it plays a role in astrocyte proliferation, dendritic cell migration, heart rate regulation, and ischemia–reperfusion injury [27]. Regulatory T cells suppress the activation and proliferation of CD4+ helper T cells, inhibit the differentiation of cytotoxic CD8+ T cells, and restrict B cell activation. However, S1P_1_ inhibits the number and function of regulatory T cells, leading to aggravation of autoimmune diseases [4].

S1P_2_ is located in both the plasma membrane and cytoplasm. It is expressed in both the CNS and the immune system and plays a pivotal role in the inhibition of apoptosis, cell proliferation, actin remodeling, and B cell localization in the follicle. Moreover, it is also implicated in the development of the cardiac, auditory, and vestibular systems. Recent studies have revealed that S1P_2_ negatively impacts myelin repair by modulating blood–brain barrier permeability, while also exerting a significant influence on demyelination [28]. Interestingly, S1P_2_, in striking contrast to S1P_1_, promotes the inflammatory response [29].

S1P_3_ is located on the plasma membrane and is expressed in certain organs (heart, lungs, kidneys, and spleen), peripheral cells, and fibroblasts, with lower expression in epithelial cells. However, its expression increases in cases of lung injury [30]. S1P_3_ plays a crucial role in cell proliferation, differentiation, apoptosis, and migration, although its function remains contentious [31]. Research suggests that S1P_3_ plays a vital role in hematopoietic stem cells and leukemia stem cells, exhibiting a protective effect against bacterial sepsis [32, 33]. Furthermore, S1P_3_ enhances the Notch signaling pathway, which is associated with retinal astrocyte proliferation. However, its role in the immune system is still unclear. Some studies indicate that S1P_3_ participates in regulating the biological functions of certain immune cells, such as dendritic cells, macrophages, and natural killer cells. Currently, most research on S1P_3_ is focused on its vascular-related aspects.

S1P_4_ receptor is expressed primarily in hematopoietic, lymphoid, and pulmonary tissues. It exerts inhibitory effects on tumor proliferation, controls the movement of immune cells, and facilitates the stimulation and maturation of dendritic cells [34, 35]. Moreover, S1P_4_ expressed on dendritic cells plays a pivotal role in enabling regulatory T cells to dampen the activity of cytotoxic T lymphocytes through its involvement in the regulation of helper T cell and interleukin 27 (IL-27) synthesis [36].

S1P_5_ receptor is present in the epidermis, oligodendrocytes, cytotoxic lymphocytes, as well as myelinated cells residing in the cerebral and splenic regions [37]. S1P_5_ exerts a neuroprotective influence through the mediation of oligodendrocyte evasion of demyelination and apoptotic processes [38]. Additionally, it is speculated that S1P_5_ may have therapeutic potential in the management of multiple sclerosis (MS) [39].

## Role of S1P and S1PRs in multiple sclerosis

**Figure 2.**
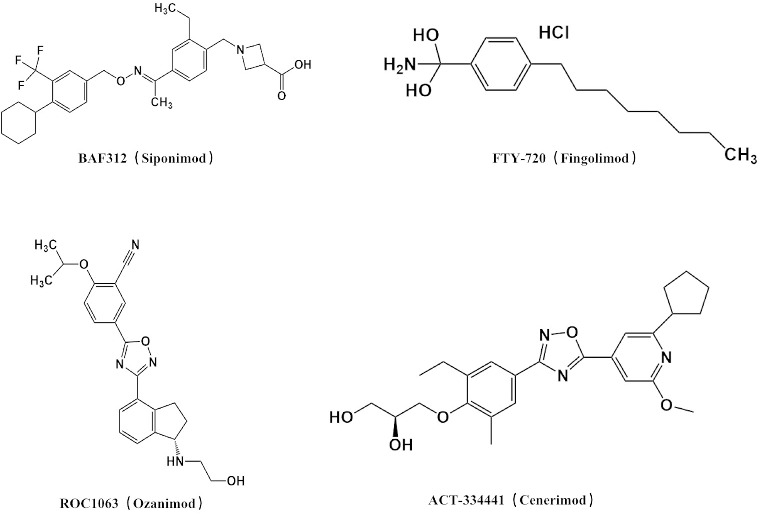
**Chemical structure of S1PR modulators.** S1PR: Sphingosine-1-phosphate receptor.

MS is an autoimmune disorder characterized by the infiltration of peripheral B and T lymphocytes into the CNS, where they attack the protective myelin sheaths surrounding neuronal axons. This results in impaired nerve conduction and the development of a disabling neurological condition, typically affecting young adults and showing an increasing incidence globally [40, 41]. Current phenotypic classifications of MS include relapsing–remitting MS, clinically isolated syndrome, radiologically isolated syndrome, primary progressive MS, and secondary progressive MS [42]. The exact pathogenesis of MS remains unclear, but it is believed that self-reactive T cells can breach the blood–brain barrier and initiate inflammatory responses against myelin antigens, leading to demyelination and neurodegeneration. Moreover, research has indicated that the activation of microglia and astrocytes within the CNS releases pro-inflammatory molecules that contribute to neuritis and secondary brain damage in MS and other CNS-related diseases [43, 44]. Of particular interest in MS is the role of S1PRs and their expression within the CNS. S1P, an extracellular signaling molecule, mediates various biological responses, including lymphocyte transport, inflammation, and vascular development, through interactions with S1PRs [45]. Interestingly, S1P is abundantly present in the CNS, and it is believed that S1P signaling modulated by S1PRs plays a crucial role in neuroinflammatory processes, which are major contributors to neurodegeneration [46, 47]. Importantly, many key neuroglial cell types (microglia, astrocytes, oligodendrocytes, and oligodendrocyte precursor cells) express S1PRs [48]. This suggests that modulating S1PRs may have therapeutic potential in the treatment of various neuroinflammatory diseases, as it can influence the activation, proliferation, and migration of glial cells. Currently, S1PR modulators are utilized to enhance systemic immune responses, with therapeutic strategies targeting S1PR initially developed for relapsing–remitting MS. Four S1P modulators (Fingolimod, Siponimod, Ozanimod, and Ponesimod) are currently approved for the treatment of MS (Table 1 and Figure 2) [49]. Notably, S1PRs S1P_1/3/5_ are expressed in neurons, astrocytes, microglia, oligodendrocytes, and oligodendrocyte precursor cells, while S1P_2_ is present in neurons, astrocytes, and microglia but not on oligodendrocyte precursor cells [50]. S1PRs are also involved in regulating lymphocyte transport, primarily through the binding of S1P_1_ on lymphocytes in MS. When S1P_1_ function is blocked, lymphocytes become sequestered in lymph nodes and the thymus, unable to respond to the S1P gradient that facilitates their exit from the lymph nodes. This reduction in circulating lymphocytes can limit the migration of inflammatory cells to the CNS [51]. Furthermore, blocking S1P_1_ inhibits the amplification of inflammatory cytokines and the recruitment of immune cells in animal models, emphasizing the goal of MS treatment to hinder immune cell infiltration into the CNS.

**Table 1:** S1PR modulators in the treatment of multiple sclerosis

**Drug**	**Receptor selectivity**	**Disease**	**Elimination T^1/2^**	**Lymphocyte recovery after drug discontinuation**
Fingolimod (FTY720)	S1PR1, 3, 4, 5	Relapsing–remitting multiple sclerosis	7 days	6 weeks
Siponimod (BAF312)	S1PR1, 5	Secondary progressive multiple sclerosis	30 h	1–10 days
Ozanimod (ROC1063)	S1PR1, 5	Relapsing–remitting multiple sclerosis, ulcerative colitis and Crohn’s disease	19–20 h	48–72 h

Table lists key protein targets and ligands in this article which are hyperlinked to corresponding entries in http://www.guidetopharmacology.org, the common portal for data from the IUPHAR/BPS Guide to PHARMACOLOGY (Southan et al., 2016) and are permanently archived in the Concise Guide to PHARMACOLOGY 2015/16. S1PR: Sphingosine-1-phosphate receptor.

Ozanimod (ROC1063) is an S1P_1/5_ agonist that exhibits 27 times greater selectivity for S1P_1_ compared with S1P_5_. Its interaction with S1PRs leads to receptor internalization and subsequent degradation via ubiquitin protease-dependent mechanisms, preventing the reassembly of receptors on the cell membrane. This mechanism results in the inhibition of lymphocyte egress from lymph nodes, leading to a decrease in absolute lymphocyte counts. However, this effect is rapidly reversible, with lymphocyte counts returning to baseline within 48–72 h upon discontinuation of the drug [52]. Ozanimod was approved by the FDA in 2020 for the treatment of relapsing MS in adults. It has undergone phase II and two phase III pivotal trials, demonstrating both efficacy and favorable tolerability [53]. In phase II trials, compared to placebo, the use of Ozanimod at doses of 0.5 or 1 mg/day resulted in fewer new or expanded T2 lesions and gadolinium-enhancing lesions on brain magnetic resonance imaging scans, with only three non-treatment-related serious adverse events reported in patients receiving Ozanimod 0.5 mg (optic neuritis, somatoform autonomic dysfunction, and cervical squamous metaplasia related to human papillomavirus). No serious infections or cardiac adverse events were reported, and no cases of macular edema were observed. The most common adverse events in the Ozanimod 1.11 mg and 12 mg groups were nasopharyngitis, headache, and urinary tract infection, relative to the placebo group [54]. Overall, Ozanimod displayed a favorable safety profile, with no significant events related to cardiovascular, pulmonary, ophthalmic, infectious, or malignant conditions. However, respiratory function should be assessed during treatment [55]. In animal models of relapsing–remitting MS, experimental autoimmune encephalomyelitis (EAE) is the most frequently used model, inducing demyelination in the nervous system. In the EAE mouse model, blocking S1P_1_ enhances neuronal survival and inhibits glial cell proliferation and demyelination. Studies have elucidated the anti-inflammatory and neuroprotective effects of Ozanimod, in which it primarily reduces glutamatergic synaptic transmission during EAE. This effect is achieved by attenuating the release of inflammatory cytokines by activated microglia and T cells [56].

Fingolimod (FTY-720), a potent anti-inflammatory agent, gained FDA approval in 2010 as the first oral medication for relapsing–remitting MS. It effectively reduces the annual relapse rate by inhibiting lymphocyte drainage from lymphoid tissues and minimizing neuroinflammation in the CNS [57]. Phase II trials demonstrated that FTY-720’s peak effectiveness against MS occurred at a suboptimal dose that induced lymphocytopenia. Interestingly, S1PRs were found to be expressed in various types of neuronal cells, leading researchers to suggest that FTY-720 might also exert direct neuroprotective effects in the brain [58]. However, phase III trials revealed serious adverse events in some patients, such as seizures, infections, and leukopenia [59]. In addition to its anti-inflammatory properties, FTY-720 reduces astrocyte proliferation and promotes oligodendrocyte differentiation within the CNS, potentially preventing brain atrophy [60]. In mice with EAE, FTY-720 decreased astrocyte proliferation and pro-inflammatory cytokine expression, thereby reducing demyelination, axonal loss, and astrocyte proliferation [61, 62]. Furthermore, the level of inhibition of astrocyte proliferation by FTY-720 correlated with its strength in inhibiting S1P_1_ function. This suggests that S1P_1_ on astrocytes plays a role in MS-related astrocyte hyperplasia and that FTY-720 requires S1P_1_ on astrocytes to exhibit its activity in an EAE mouse model [63]. Moreover, FTY-720 inhibits the release of pro-inflammatory chemokines, cytokines, and neurotoxic factors, such as C-X-C motif chemokine 5, induced by lipopolysaccharide stimulation in astrocytes and microglia [64]. In an EAE mouse model, FTY-720 derivatives ST-1893 and ST-1894 caused severe lymphopenia and alleviated symptoms [65]. Another study focused on novel butterfly derivatives of FTY-720, namely, ST-1505 and ST-1478, both agonists of S1P_1/3_ and S1P_1/5_ receptors, respectively. These derivatives show promise as new drugs for relapsing–remitting MS and other immune diseases. Additionally, they possess histamine H3 receptor antagonistic properties and may improve cognitive impairment in relapsing–remitting MS. This suggests that S1P_1_-selective compounds could potentially be more effective than prodrugs in treating relapsing–remitting MS [66].

Siponimod (BAF312) exhibits strong affinity for S1P_1_ and S1P_5_ receptors expressed by endothelial cells that constitute the blood–brain barrier and prevent lymphocytes from entering the CNS. By concurrently modulating S1P_1/5_, Siponimod enhances the properties of the blood–brain barrier, thereby offering additional effectiveness in treating secondary progressive MS [67]. Furthermore, Siponimod induces a balanced state of anti-inflammation and suppression in the immune system by enriching regulatory T cells, transitional regulatory B cells, and B1 subpopulations. These changes potentially contribute to the clinical efficacy of Siponimod in the treatment of secondary progressive MS [68]. In a phase III clinical study spanning over five years, continuous administration of the S1P modulator Siponimod resulted in a significant reduction in disability progression, rate of cognitive deterioration, and maintenance of low annual relapse rates, as well as reductions in total and localized brain atrophy and inflammatory activity in patients with secondary progressive MS. These improvements were particularly prominent in patients who started Siponimod treatment early and maintained its use [69]. Another phase III clinical trial evaluating Siponimod in secondary progressive MS showed that adverse events occurred in 89% of patients (975 of 1099) who received Siponimod treatment. These events included lymphopenia, elevated hepatic transaminase concentrations, bradycardia and bradyarrhythmia at the start of treatment, macular edema, hypertension, varicella-zoster reactivation, and convulsions, which occurred more frequently in the Siponimod group [70]. Siponimod also exerts an effect on astrocytic hyperplasia and microgliosis. In mice with EAE, administration of Siponimod attenuated the proliferation of astrocytes and microglia, and reduced lymphocyte infiltration, thereby dampening the inflammatory response [71]. It was also observed that Siponimod decreased macrophage infiltration and microglia activation in the spinal cord of EAE mice [38], and in mice with traumatic brain injury, it decreased the activation of CD4+ and CD8+ T cells, as well as the extent of injury [72]. The importance of S1P in the CNS is progressively being recognized, and the multifaceted effects of FTY-720 and other drugs that target SPHK suggest that this approach should be considered for the treatment of other neurodegenerative diseases.

## Role of S1P and S1PRs in systemic lupus erythematosus

Systemic lupus erythematosus (SLE) is a chronic autoimmune disease characterized by various clinical phenotypes. While the exact cause remains unknown, it is believed that a combination of genetic and environmental factors contributes to its development [73]. SLE is characterized by immune system dysfunction and the persistent presence of an inflammatory environment, often resulting in damage to multiple organs and, ultimately, the death of the patient. However, the pathogenesis of SLE remains unclear, and available treatment options are limited. The S1PR, a recognized regulator of lymphocyte transport with potent immunomodulatory functions, is closely associated with SLE and other autoimmune diseases. Elevated levels of S1P have been observed in the serum of SLE patients, which leads to an increase in the number of lymphocytes in the peripheral blood and the infiltration of inflammatory cells into organs [74]. In mouse models of SLE, increased S1P levels have been shown to exacerbate disease activity and organ damage. In patients with SLE, the expression of various pro-inflammatory cytokines is increased, leading to an increase in S1P production. Conversely, elevated levels of S1P further aggravate the inflammatory state by activating inflammatory signaling, recruiting immune cells, and promoting the release of pro-inflammatory mediators [75].

Cenerimod (ACT-334441) is an orally administered modulator of the S1P_1_ receptor. Unlike many S1PR modulators, which often induce bronchoconstriction or vasoconstriction, Cenerimod does not cause these side effects in the non-clinical setting. In an animal study involving SLE and Sjögren’s syndrome mice, Cenerimod was found to improve both systemic and organ-specific autoimmunity, resulting in reduced inflammation and preserved organ function [76]. A phase II clinical trial investigated the effects of Cenerimod on SLE activity and circulating lymphocytes in patients with mild to moderate SLE. The trial focused on patients with a SLEDAI-2K score of at least two for musculoskeletal or mucosal manifestations, a history of antinuclear or anti-double-stranded DNA antibodies, or a positive serologic test at screening. The findings from this trial suggest that Cenerimod has the potential to treat SLE in patients, while also ensuring safety [77]. Studies have demonstrated that Cenerimod significantly improves both systemic and organ-specific pathology and inflammation in SLE mice. For example, it reduces levels of plasma anti-double-stranded DNA and immunoglobulin, decreases blood B and T lymphocyte counts, and diminishes circulating and organ-infiltrating lymphocytes. Additionally, Cenerimod decreases proteinuria and improves survival [78]. Similarly, research has shown that Ozanimod, a small molecule modulator of S1P_1_ and S1P_5_ receptors, attenuates chronic inflammation and renal pathology in SLE mice.

### Role of S1P and S1PRs in lupus nephritis

Lupus nephritis is the most prevalent complication of SLE, and its progression indicates a poor prognosis and a high mortality rate [79]. S1P regulates the production of inflammatory mediators, and elevated S1P levels have also been associated with the development of SLE [80]. One study demonstrated that S1P expression was significantly increased in the serum of SLE patients with lupus nephritis activity [81]^.^ Similarly, mice with lupus nephritis had elevated S1P expression in their serum [82]. These findings suggest that S1P and its receptors play a role in the development of lupus nephritis. Moreover, S1P_3_ has been observed in the renal cortex, outer medulla, and inner medulla [83]. Activation of the S1P/S1P_3_ signaling pathway suppresses the number and function of regulatory T cells. Transplantation of regulatory T cells before clinical disease onset has been shown to reduce kidney injury progression and mortality in mice predisposed to lupus [84]. FTY720 sequesters circulating lymphocytes within lymph nodes, thereby decreasing the infiltration of autoimmune cells (T and B lymphocytes) and inflammatory factors such as IL-6 and TNF-α from the bloodstream and inflammatory tissues into target organs. This leads to the relief of SLE symptoms. Administration of FTY720 to lupus mice has been found to reduce renal injury and alleviate end-stage glomerular inflammation [85]. Additionally, FTY720-treated lupus mice exhibit decreased production of anti-double-stranded DNA in the glomerulus and reduced accumulation of IgG, which consequently inhibits the progression of lupus nephritis [86].

### Role of S1P and S1PRs in neuropsychiatric lupus

Neuropsychiatric SLE is a severe complication of SLE, in which patients commonly exhibit nonspecific symptoms like headache and cognitive impairment. However, they may also develop more destructive symptoms, such as memory loss, seizures, and stroke, with a mortality rate second only to that of lupus nephritis [87]. The central and peripheral nervous systems of SLE patients can be targeted, leading to neurological or psychiatric symptoms that contribute to cognitive impairment and depression. Although much remains unknown concerning the pathogenesis of neuropsychiatric SLE [88], one study discovered that the disruption of the intact blood–brain barrier is a primary factor in disease progression [89]. Cenerimod, a selective S1PR modulator, has been found to directly impact brain endothelial cells, reducing blood–brain barrier permeability and achieving symptom relief [90]. Moreover, Cenerimod also plays a neuroprotective role. In lupus mice, administration of the S1P inhibitor FTY-720 mitigated neurobehavioral deficits, including spatial behavior and depressive behavior, while also reducing cortical, hippocampal, and amygdalar neuronal damage. Additionally, it suppressed inflammatory expression in astrocytes and endothelial cells, although cytokine levels in the cerebral cortex and hippocampus were not significantly decreased. Notably, IgG deposition in the mouse brain was not affected [91]. Similarly, FTY-720 has been shown to significantly attenuate impulsive and depressive-like behavior, reduce levels of inflammatory cytokines, and decrease the infiltration of T cells and neutrophils into the brain parenchyma in lupus mice. Furthermore, FTY720 acts on endothelial cells in the brains of lupus mice by inhibiting the expression of multiple signaling pathways and reducing the permeability of the blood–brain barrier, resulting in decreased central levels of IgG and albumin [90].

## Role of S1P and S1PRs in rheumatoid arthritis

Rheumatoid arthritis (RA) is the most prevalent systemic autoimmune rheumatic disease, affecting approximately 1% of the population with a male-to-female ratio of 1:2.5. The disease can manifest at any age but is more commonly observed in middle and old age, with its occurrence increasing with age [92]. The primary pathological changes in RA include synovial inflammation and proliferation, as well as the production of autoantibodies (rheumatoid factor and anti-citrullinated protein antibodies). These autoantibodies contribute to the damage and disability of cartilage and bone [93]. The synovial microenvironment primarily involves fibroblast-like synoviocytes (FLS) and vascular endothelial cells. FLS promotes the release of inflammatory factors and induces vascular endothelial growth factor (VEGF) production, thereby stimulating endothelial progenitor cells and promoting angiogenesis and RA development [94]. The S1P/S1PR axis plays a significant role in RA, as S1PRs were found to be elevated in RA model mice. RAFLS produced IL-6 in response to S1P stimulation, and S1P_3_ expression levels increased with pre-activation by tumor necrosis factor alpha (TNF-α). An increase was also observed in IL-6 and matrix metalloproteinase 3 (MMP3) production induced by S1P3 [95]. Inhibition of the S1P/S1PR (S1P_1_/S1P_2_/S1P_3_/S1P_4_) signaling pathway can suppress the proliferation, migration, pro-inflammatory, and apoptosis-promoting activities of RAFLS. Additionally, IL-17 expression can be reduced [96, 97]. Recent studies have demonstrated the expression of S1P and S1P_1_ receptors in the synovium in RA. Synovitis is the primary cause of elevated cytokine levels (TNF-α, IL-1, IL-6, IL-1β), and these inflammatory factors induce SPHK1 in a manner dependent on extracellular signal-regulated kinase signaling [98, 99]. Consequently, high levels of S1P are produced, which partly explains the presence of inflammatory tissue [100]. Furthermore, S1P signaling promotes synovial cell proliferation and induces the production of prostaglandin E2 by upregulating the expression of cyclooxygenase-2 in response to inflammatory cytokines [101]. Inflammatory cytokines also induce the elevation of MMPs, thereby activating osteoclasts and creating a vicious cycle. Other studies have demonstrated that S1P, through its receptor S1P_1_, enhances the expression of receptor activator of NF-κB ligand (RANKL), leading to the production of inflammatory cytokines and bone erosion [102].

In animal experiments, FTY720 was observed to mitigate the expression of IL-6 and TNF-α in the synovial membrane of mice with RA [103]. Based on animal experiments, gardenia glycosides were discovered to deplete S1P or impede the activation of SPHK1, blocking the interaction between FLS and vascular endothelial cells [104]. Furthermore, recent animal experiments have revealed that gardenia glycosides can inhibit the activation of the S1P/S1PR1 signaling pathway through SPHK1, leading to reduced expression of VEGF, inhibition of neoangiogenesis, diminished production of synovial tissue, alleviation of bone erosion, and effective alleviation of RA symptoms [105]. Additionally, gardenia glycosides can also suppress the SPHK1/S1P pathway to alleviate vascular endothelial cells stimulation caused by FLS, inhibit angiogenesis, and attain therapeutic effects [106]. Recently, S001PR930 and the S1PR1 modulator IMMH1 (SYL4) inhibited the progression of arthritis in Sprague-Dawley rats, as evidenced by reduced hind paw swelling and reduced arthritis index, alongside decreased levels of pro-inflammatory cytokines and chemokines in affected joints [107]. Despite these promising outcomes observed in animal studies, no ongoing clinical studies have been conducted on individuals with RA. Therefore, for future RA treatments, in addition to conventional approaches, such as non-steroidal anti-inflammatory drugs, corticosteroids, disease-modifying antirheumatic drugs, and biologics, downregulation of S1P may be considered as a means to achieve effective RA control.

## Role of S1P and S1PRs in systemic sclerosis

Systemic sclerosis (SSc), a rare and intricate systemic autoimmune rheumatic disease, is characterized by immune irregularities, vascular lesions, and fibrosis. Primarily affecting the skin, gastrointestinal tract, lungs, kidneys, and heart [108], SSc presents a mortality risk of up to 20% through its complication of interstitial lung involvement. Following an interstitial lung diagnosis, primary or secondary pulmonary hypertension occurs in 8%–12% of patients, leading to a 25% mortality rate within 3 years [109]. Consequently, urgent exploration into novel therapies is imperative to mitigate the morbidity and mortality associated with SSc. Studies have revealed aberrant activation and differentiation of B cells in SSc patients [110]. Moreover, T cell-derived cytokines exhibit anomalies in SSc patients [111], highlighting the significance of lymphocyte regulation in SSc treatment. The S1P/S1PRs signaling pathway has been implicated in the pathogenesis of SSc. Animal models of fibrosis (lung, liver, and kidney) have demonstrated S1P involvement in fibrotic development, and elevated serum S1P levels have been observed in SSc patients due to increased activation of platelet aggregation that occurs during SSc lesions. This increased S1P binding to its receptors, S1P_2_ and S1P_3_, induces vasoconstriction, promotes vascular smooth muscle cell proliferation, and stimulates neointima formation, ultimately causing vasospasm and Raynaud’s phenomenon [112]. Skin tissues of SSc patients express S1P_1/2/3/5_, with reduced expression levels of S1P_1_ and S1P_2_ receptors and increased levels of S1P_3_ receptors due to altered distribution of S1P isoforms in fibroblasts. The impact of S1P_5_ on inflammatory and pro-fibrotic processes remains unknown [113, 114]. Furthermore, S1PRs play a role in facilitating the migration of lymphocytes from the thymus or secondary tissues into the bloodstream [115], and obstructing this pathway can impede the expression of inflammatory cytokines in SSc patients, leading to a deceleration of inflammation and fibrosis.

FTY720 has been shown to potentially reduce the upregulation of type I interferon in SSc by plasmacytoid dendritic cells [116]. FTY720 has also demonstrated the ability to decrease T helper 17 cell differentiation and functionality, which in turn promotes fibrosis by increasing the abundance of T helper 17 cells and plasmacytoid dendritic cells in a bleomycin-induced scleroderma mouse model [117]. The increased presence of these two cell types has also been observed in SSc patients [118, 119]. Cenerimod, an S1P_1_ receptor modulator, reduces blood lymphocytes, and early administration of Cenerimod has been proven effective in reducing skin and lung fibrosis in bleomycin-induced SSc mice. This reduction is accompanied by a decrease in IL-6 and collagen deposition [120]. FTY720 is believed to exert its immunomodulatory effects by reducing blood lymphocytes. However, long-term administration of FTY720 exacerbates bleomycin-induced SSc by causing vascular leakage and intra-alveolar coagulation, ultimately leading to fibrosis and death in SSc mice [121]. Therefore, selective S1PR receptor modulators are more suitable for treating SSc. Studies have shown that Cenerimod administration inhibits the infiltration of CD4+ T cells, CD8+ T cells, and CD11+ B cells into inflamed skin, increases the number of regulatory T cells in the spleen and skin, and reduces the expression of extracellular matrix and fibroblast cytokines in the skin in a study on bleomycin-induced fibrosis models. It also attenuates skin and lung fibrosis [120]. Therefore, Cenerimod holds promise as a treatment option for SSc patients and could potentially slow down the progression of the disease. Multiple S1PRs may play a role in the pathophysiological changes of SSc, affecting various immune, vascular, and cellular aspects. Only through a deeper understanding of these mechanisms can we develop more effective and rational therapeutic approaches.

## Role of S1P and S1PRs in bone immunology

The connection and interaction between the skeletal system and the immune system are referred to as osteoimmunology. It has become an indispensable theoretical foundation for understanding the pathophysiological mechanisms of certain diseases, such as RA, ankylosing spondylitis, and osteoarthritis. S1P and S1PRs play a role in both the immune system and bone remodeling. The role of S1P/S1PR1 signaling in bone remodeling can directly target osteoclastogenesis and osteogenesis, due to the significant role of S1P/S1PR1 signaling in inflammation [122], which is considered a catalyst for inflammation-induced osteoclastogenesis. Moreover, the functionality and polarization of inflammatory cells in the adaptive immune system (T cell subsets) and innate immune cells (macrophages) are also regulated by this signaling axis. This suggests that the S1P/S1PR1 signaling axis can indirectly regulate bone remodeling through the modulation of the immune system [123]. Additionally, S1P controls the migration of osteoclast precursor cells between the bloodstream and the bone marrow, partly mediated by S1PRs expressed on the surface of osteoclast precursor cells (S1P_1_ and S1P_2_) [124]. Animal studies have indicated that inhibition of S1P_2_ function alters the dynamics of osteoclast precursor cell migration, thereby alleviating osteoporosis [125]. However, inflammatory cytokines, such as IL-6, IL-17, and IL-1β, play important roles in bone loss. IL-6 increases the number of osteoclast precursor cells by upregulating S1P_2_, contributing to inflammation-induced bone loss. In a mouse model of RA, elevated IL-6 expression upregulates S1P_2_, thereby increasing the number of osteoclast precursor cells. These findings suggest the need to control the expression of inflammatory cytokines in the treatment of RA to prevent systemic bone loss [126]. S1P promotes IL-1β expression in osteoblasts through the S1P_1_ receptor and JAK/STAT3 signaling pathway. Knocking out SPHK1 reduces IL-1β expression in osteoblasts [101], and IL-17 enhances TNF-α-induced IL-6 synthesis in osteoblasts via activation of the p38 MAP kinase [127]. In addition to IL-17 stimulation, S1P can also promote IL-6 expression in osteoblasts through the phosphoinositide 3-kinases (PI3K), MEK/ERK, and NF-κB signaling pathways [128]. The elevation of inflammatory cytokines induces VEGF production, exacerbating bone loss. Therefore, IL-6 receptor antibodies alleviate systemic bone loss and reduce the number of osteoclast precursor cells in the tibial bone marrow by downregulating S1P_2_ [126]. Similarly, symptom relief can also be achieved by blocking IL-17 expression and reducing IL-6 synthesis. Another study suggests that active vitamin D regulates the migration behavior of osteoclast precursor cells in the circulation, which helps to limit osteoclastogenic bone resorption [129]. Osteoclasts, a special subpopulation of cells with bone-resorbing ability, play a critical role in maintaining normal bone homeostasis, degrading old bone, and promoting the formation of new bone. The expression of SPHK and the production of S1P in osteoclasts stimulate the migration of mesenchymal stem cells by activating kinase signaling pathways [130]. S1P also promotes osteoblastogenesis. Under RANKL stimulation, S1P expression is enhanced in osteoclast precursors, and by binding to S1PRs on the surface of osteoblasts, S1P promotes the migration and survival of osteoblasts. Subsequently, S1P-activated osteoblasts upregulate RANKL expression, which stimulates the migration, fusion, activation, and survival of osteoclasts, leading to increased osteoclastogenesis [131, 132]. Osteoclasts participate in cartilage damage in osteoarthritis via the S1P/S1P_2_ pathway. The activity of SPHK1 increases with osteoclast differentiation, and injecting sphingosine analogs into the joint cavity of mice reduces S1P levels and alleviates bone arthritis [133]. Furthermore, FTY720 alleviates ovariectomy-induced osteoporosis in mice by promoting the recirculation of cell populations containing osteoclast precursors and reducing the number of mature osteoclasts attached to the bone surface [124]. Studies have also shown that SPHK inhibitors such as FTY720 combined with zoledronate can reduce inflammatory bone erosion and inhibit osteoclast differentiation [134]. The levels of S1P generated by SPHK phosphorylation are positively correlated with bone formation markers. By inhibiting S1P lyase to increase S1P levels, there is a significant increase in the quality and strength of bone formation [135]. Regulating the signaling pathways of SPHK1/S1P/S1P_1/2_ holds promise as an effective approach for treating bone metabolic disorders, such as osteoporosis, RA, and osteoarthritis.

## The role of S1P and S1PRs in Sjogren’s syndrome

Sjogren’s syndrome is a systemic autoimmune disease characterized by the gradual destruction of the lacrimal and salivary glands due to an inflammatory process, resulting in dryness of the eyes and mouth [136]. The affected glands are heavily infiltrated by lymphocytes, with T lymphocytes being more prominent in mild cases, while B lymphocytes are most representative in severe cases [137]. Previous studies have demonstrated the involvement of the S1P/S1P_1_ axis in the pathogenesis of the disease [138]. Cellular studies have shown the expression of S1P_1/2/3/4_ receptors, as well as SPHK1 and SPHK2, in human submandibular gland cells, and further evidence suggests that S1P triggers the Ca^2+^ signaling pathway and apoptotic pathway in normal submandibular cells, thus affecting the progression of Sjogren’s syndrome by interfering with S1P_1/2/3/4_. Additionally, S1P induces the expression of Fas and IL-6, which are related to the pathology of Sjogren’s syndrome [139]. Clinical research has indicated that the S1P signaling pathway may regulate the autoimmune phenotype of Sjogren’s syndrome through immune cells and epithelial cells. Lymphocytes in the peripheral blood of primary Sjogren’s syndrome patients are significantly higher compared to healthy individuals [138], and S1P plays a major role in lymphocyte migration through interaction with its receptors, S1PRs. Cenerimod can inhibit lymphocyte entry into the circulation, reduce immune infiltration of salivary glands, alleviate salivary gland inflammation, and relieve symptoms in viral-induced salivary gland C57 mouse models, among others. In the chronic sialadenitis mouse model, oral administration of Cenerimod can reduce salivary gland inflammation, as well as T cells and proliferating plasma cells in ectopic lymphoid structures within the salivary gland, leading to a decrease in disease-associated autoantibodies [140]. These findings suggest that future treatments for Sjogren’s syndrome may consider the use of S1P_1/2/3/4_ receptor inhibitors to control its progression.

### Role of S1P and S1PRs in pulmonary hypertension associated with connective tissue disease

Pulmonary arterial hypertension (PAH) is a prevalent and critical complication of connective tissue diseases, characterized by progressive alterations in pulmonary vascular function and structure. The primary pathophysiological manifestations of PAH involve increased vasodilatory tone, impairment in vascular endothelial function, and structural remodeling in the small- and medium-sized pulmonary arteries. These changes give rise to increased pulmonary vascular resistance, right-sided heart hypertrophy, and ultimately, right ventricular failure [141, 142]. The development of connective tissue disease-associated PAH is linked to autoimmunity against G protein-coupled receptors, while the involvement of S1P/S1PR signaling in the pathogenesis of PAH has also been reported [143]. S1P is expressed at elevated levels in the human lung [144] and facilitates autophagy activation by accelerating the upregulation of TNF receptor-related factor 2-mediated BECN1 and ubiquitination. Subsequently, cadherin-1 degradation and proliferation of pulmonary artery smooth muscle cells (PASMCs) occur [145]. Another signaling pathway, the Smad2/3/SPHK1/S1P/Notch3 pathway, mediated by tumor growth factor beta 1 (TGF-β1), also induces PASMC proliferation [146]. Therefore, these aforementioned pathways hold potential as targets for the prevention of PAH. Not only does SPHK/S1P mediates the proliferation of PASMCs and remodeling of the pulmonary artery [141] but S1PRs also regulate the proliferation of PASMCs [147], potentially due to the high expression of S1PRs in these cells. Inflammatory cells and PASMCs represent two major cell populations involved in the pathogenesis of PAH. The processes of inflammation and angiogenesis contribute to vascular remodeling and the excessive proliferation of smooth muscle cells, which are characteristic of severe PAH [148, 149]. S1P/S1PR signaling plays a crucial role in maintaining vascular dynamic homeostasis. Impairment in S1P/S1PR signaling leads to vasoconstriction, vascular remodeling, and endothelial dysfunction, thereby increasing pulmonary vascular resistance and mean pulmonary arterial pressure, ultimately culminating in PAH. Early blockade of S1P_2_, inhibition of Notch3, silencing of STAT3, miRNA-135b, or YAP all attenuate S1P-induced proliferation and vascular remodeling of PASMCs, thus preventing the development of PAH [150]. Animal experiments have confirmed the finding of increased SPHK1 protein levels and S1P production in the lung tissue of animal models of idiopathic pulmonary hypertension [151], and inhibition of SPHK1, S1P, or S1P_2_ slowed down the progression of PAH induced by chronic hypoxia in animals or wild lily pads [152]. However, the treatment of PAH remains challenging. Animal experiments have shown that resveratrol effectively inhibits the progression of PAH by blocking SPHK1/S1P-mediated NF-κB activation [151]. Similarly, serpentine inhibits highly expressed S1P by regulating the metabolic enzyme SPHK1 in PAH animals. The drug also downregulates miRNA-21, Akt phosphorylation, and mTOR phosphorylation, thereby inhibiting SPHK1 expression. These findings present a potential treatment for connective tissue disease-associated PAH [153]. Stem cell therapy has emerged as a promising therapeutic modality for PAH, with human bone marrow mesenchymal stem cells pretreated with S1P enhancing the therapeutic effect on PAH. Additionally, S1P-induced mesenchymal stem cells enhance the anti-inflammatory and angiogenic activity of fibroblasts in culture. Furthermore, in wild lily bine-induced PAH animal experiments, cells stimulated with S1P significantly reduce the elevation of right ventricular systolic pressure [154]. By optimizing stem cell therapy in the future and combining it with potential targeted drugs for PAH, novel treatment approaches may be developed for this condition.

## Conclusion

The pleiotropic bioactive lipid S1P and its S1PRs play crucial roles in the development and functioning of the immune, cardiovascular, neurological, and skeletal systems. The significance of the S1P/S1PR axis in autoimmune diseases has paved the way for innovative treatments in this domain. Initially, S1P was discovered to regulate the efflux of lymphocytes from lymphoid tissues. By modulating the S1P/S1PR pathway, autoimmune diseases can be attenuated in terms of immune and inflammatory responses. Further research has shed light on the neuroprotective effects of this pathway through its interaction with S1PR on neuronal cells. The advent of S1PR modulators has revolutionized the treatment landscape not only for multiple sclerosis but also for other autoimmune diseases, such as systemic lupus erythematosus, rheumatoid arthritis and systemic sclerosis. Nonetheless, the quest for an optimal treatment for numerous autoimmune diseases persists. As a result, early diagnosis becomes of paramount importance, as it enables clinicians to tailor patient-specific treatments.

Given that the mechanism of the S1P/S1PR signaling pathway in autoimmune disorders is still under intensive investigation, the conclusions presented in this review may exhibit a slight bias. Nevertheless, this review has the potential to enhance clinicians’ comprehension of the advancements made in the field of S1P/S1PR signaling pathway in autoimmune diseases. Further extensive research into the functionality of S1P signaling in various diseases, combined with the development of more targeted drugs alongside their optimal administration systems, will introduce novel therapeutic alternatives. The pursuit of more specific pharmacological agents and the establishment of refined drug delivery systems will yield innovative therapeutic strategies. The judicious manipulation and utilization of S1PR modulators, coupled with a comprehensive understanding of the crucial role played by the S1P/S1PRs axis in autoimmune diseases, present themselves as promising avenues for future exploration.
